# Are All Anti-Angiogenic Drugs the Same in the Treatment of Second-Line Metastatic Colorectal Cancer? Expert Opinion on Clinical Practice

**DOI:** 10.3389/fonc.2021.637823

**Published:** 2021-05-10

**Authors:** Eleonora Lai, Stefano Cascinu, Mario Scartozzi

**Affiliations:** ^1^ Medical Oncology Unit, University Hospital and University of Cagliari, Cagliari, Italy; ^2^ Oncologia Medica, Università Vita-Salute, IRCCS Ospedale San Raffaele, Milano, Italy

**Keywords:** aflibercept, angiogenesis, biomarkers, metastatic colorectal cancer, second-line therapy, anti-VEGF

## Abstract

Targeting tumor-driven angiogenesis is an effective strategy in the management of metastatic colorectal cancer (mCRC); however, the choice of second-line therapy is complicated by the availability of several drugs, the occurrence of resistance and the lack of validated prognostic and predictive biomarkers. This review examines the use of angiogenesis-targeted therapies for the second-line management of mCRC patients. Mechanisms of resistance and anti-placental growth factor agents are discussed, and the role of aflibercept, a recombinant fusion protein consisting of portions of human vascular endothelial growth factor receptor (VEGFR)-1 and VEGFR-2, is highlighted. The novel mechanism of action of aflibercept makes it a useful second-line agent in mCRC patients progressing after oxaliplatin-based chemotherapy, as well as in those with resistance after bevacizumab.

## Introduction

Colorectal cancer (CRC) is the fourth most common tumor and it stands at the second place for cancer death worldwide (1). Almost 20% of CRC patients are diagnosed at an advanced stage and approximately a further 20% develop metastases later in life (2). Nowadays, it is well known that tumor-driven angiogenesis plays a crucial role in CRC growth and metastatic spread (3–15).

Tumor angiogenesis leads to the formation of new blood vessels through a very complex and coordinated process (16–19). Physiologic angiogenesis is a well-controlled event, regulated *via* the balance of pro- and antiangiogenic factors (19). Among these, the vascular endothelial growth factor (VEGF) signaling represents a key pathway and it comprises a family of five secreted proteins [VEGF-A, VEGF-B, VEGF-C, VEGF-D, and placental growth factor (PlGF)] and three receptor tyrosine kinases (RTK): VEGF receptor (VEGFR)-1, VEGFR-2, and VEGFR-3 (19, 20). VEGFR-1 [also named Fms-like tyrosine kinase-1 (FLT1)] and VEGFR-3 [also named Fms-related tyrosine kinase 4 (FLT4)] have been shown to be involved in tumor progression and metastasis in CRC, and VEGFR-2 (also named fetal liver kinase FLK1/KDR) has been implicated in endothelial cell survival, proliferation and migration (21, 22). Furthermore, PlGF induces angiogenesis *via* several mechanisms, both directly and indirectly (23–25). Consequently, angiogenesis is one of the most important therapeutic targets for the treatment of metastatic CRC (mCRC) and a consistent number of agents targeting the angiogenesis pathway have been developed and are now available for mCRC patients across different lines of treatment (2, 26).

The aim of this review is to explore the role of anti-angiogenesis strategies in the second-line management of mCRC, including mechanisms of resistance and the use of anti-PlGF agents. In particular, individualized treatment is discussed. Moreover, the role of aflibercept, a recombinant fusion protein consisting of portions of human VEGFR-1 and -2 (27), is emphasized, in order to discuss the results and potential benefits showed by this innovative agent with a unique mechanism of action.

## Management of mCRC: Targeting the Angiogenesis in the Second-Line Setting

Medical treatment of mCRC is mainly palliative, aimed at slowing disease progression, prolonging survival and maintaining quality of life (QoL), even if in some specific cases the curative aim cannot be excluded (e.g. single resectable metastatic site) (28). Antiangiogenic agents are among the most effective drugs for the treatment of mCRC, both in first and second-line setting; they are recommended in combination with fluoropyrimidine-based chemotherapy plus oxaliplatin and/or irinotecan (29–31). Antiangiogenic agents currently approved for the treatment of mCRC in Europe are shown in **Table 1**.

**Table 1:** Antiangiogenic agents currently approved for the treatment of mCRC in Europe.

Agent	Mechanism of action	Indications	Ref
Bevacizumab	VEGF inhibitor	mCRC in combination with fluoropyrimidine-based chemotherapy	(32)
Aflibercept	A soluble decoy receptor that inhibits the binding and activation of VEGF-A, VEGF-B and PlGF	In combination with FOLFIRI in patients with mCRC that is resistant to, or has progressed after, an oxaliplatin-containing regimen	(27)
Ramucirumab	Anti-VEGFR-2 monoclonal antibody	In combination with FOLFIRI in mCRC patients with disease progression on or after bevacizumab, oxaliplatin and a fluoropyrimidine	(33)
Regorafenib	Multi-kinase inhibitor that targets angiogenesis (VEGFR-1, -2, -3, TIE2), as well as oncogenesis (KIT, RET, RAF-1, BRAF, BRAF V600E), metastasis (VEGFR3, PDGFR, FGFR) and tumor immunity (CSF1R)	mCRC after previous treatment with fluoropyrimidine-, oxaliplatin- and irinotecan-based chemotherapy, anti-VEGF and -EGFR therapies and chemotherapy	(34)

BRAF, v-raf murine sarcoma viral oncogene homolog B1; CSF1R, colony-stimulating factor 1 receptor; EGFR, epidermal growth factor receptor; FGFR, fibroblast growth factor receptor; FOLFIRI, folinic acid (leucovorin), 5-fluorouracil and irinotecan; KIT, v-kit Hardy-Zuckerman 4 feline sarcoma viral oncogene homolog; mCRC, metastatic colorectal cancer; PDGFR, platelet-derived growth factor receptor; PlGF, placental growth factor; RAF-1, v-raf-1 murine leukemia viral oncogene homolog 1; RET, rearranged during transfection; VEGF(R), vascular endothelial growth factor (receptor).

The choice of second-line treatment is individually tailored depending the therapeutic scheme received in the first-line setting (29, 31, 35–38) and its outcome and how well it was tolerated, patient fitness and clinical characteristics plus their tumor biologic and molecular features, especially rat sarcoma viral oncogene homolog (RAS)/v-raf murine sarcoma viral oncogene homolog B1 (BRAF) mutation status (2, 29, 31, 39, 40). Second-line approved angiogenesis-targeted options include bevacizumab, aflibercept and ramucirumab (41–46), which all demonstrated an increase in overall survival (OS) in the second-line setting in phase III trials (**Table 2**). Unfortunately, these trials included different patient populations and no head-to-head randomized phase III trials have been conducted to compare these three agents (54). Moreover, no randomized studies have been conducted to evaluate the best treatment sequence after first-line anti-EGFR and anti-VEGF (2, 39, 40).

**Table 2:** Main clinical trials of anti-angiogenic agents in the second-line setting.

Anti-angiogenic agent	Trial	Study design	Study population	Study arms	Stratification	Pts (N)	Primary EP	Efficacy	Safety	Ref
Bevacizumab	E3200	R, OL, Phase III	mCRC; PD after fluoropyrimidine and irinotecan	FOLFOX4 –bevacizumab vs	1. prior radiotherapy (yes vs no)	829	OS	FOLFOX4-bevacizumab vs FOLFOX4:	FOLFOX4-bevacizumab vs FOLFOX4:	(41)
				FOLFOX4 vs	2. ECOG PS			**mOS** 12.9 m (95%CI: 12.09–14.03) vs 10.8 m (95%CI: 10.12–11.86)	Neuropathy	
				single agent bevacizumab^1^	(0 vs 1 or 2)			(HR: 0.75; p=0.0011)	G3:16% vs 8.8%	
								**mPFS** 7.3 m vs 4.7 m	G4: 0.3% vs 0.4% Hypertension	
								(HR: 0.61; p<0.000)	G3: 5.2 vs 1.4	
								**RR** 22.7% vs 8.6%	G4 1% vs 0.4%	
								(p<0.0001)	Bleeding	
									G3: 3.1% vs 0.4%	
									G4: 0.3% vs 0%	
									Vomiting	
									G3: 8.7% vs 2.8%	
									G4: 1.4% vs 0.4%	
	ML18147 (TME)	R, OL, Phase III	mCRC; PD <3 m after stopping 1^st^-line bevacizumab +CT (including fluoropyrimidine + oxaliplatin/irinotecan).	Switch from oxaliplatin-based 1^st^ line CT→2^nd^ line irinotecan-based CT ± bevacizumab or	1. first-line CT backbone (irinotecan-based vs oxaliplatin-based)	820	OS	CT-bevacizumab vs CT:	G≥3 AEs	(42, 47)
			Pts with 1st-line PFS<3m and who had been treated <3m (consecutive) with 1st-line bevacizumab were excluded.	Switch from 1^st^ line irinotecan-based CT → 2^nd^ line oxaliplatin-based CT ± bevacizumab	2. first-line PFS (≤9 m vs >9 m)			**mOS** 11.2 m vs 9.8 m	CT-bevacizumab vs CT:	
					3. time from last bevacizumab dose			(HR: 0.81; 95%CI: 0.69–0.94; p=0.0062)	Neutropenia 16% vs 13%	
					(≤42 days vs >42 days)			CT-bevacizumab vs CT:	Diarrhea 10% vs 8%	
					4. ECOG PS			**mPFS** 5.7 m vs 4.1 m	Fatigue 3% vs 2%	
					(0-1 vs 2)			(HR: 0.68; 95%CI: 0.59–0.78; p<0.0001)		
								**RR** 5% vs 4%		
								**OS** from the start of first-line treatment: 23.9 m vs 22.5 m		
								(HR: 0.90; 95%CI: 0.77–1.05; p=0.17)		
								CT-bevacizumab vs CT:		
								**DCR** 68% vs 54%		
								(p < 0.0001)		
	BEBYP	R, OL, Phase III	mCRC; PD after or during 1st-line CT with bevacizumab	Second-line CT-bevacizumab vs second-line CT	1. ECOG PS (0 vs 1–2)	185^2^	PFS	CT-bevacizumab vs CT:	No statistically significant difference in AE incidence between the two arms	(43)
					2. CT-free interval (>3 vs ≤3 m)			**mOS** 15.5 m vs 14.1 m		
					3. second-line CHT regimen (FOLFIRI vs mFOLFOX-6)			(aHR: 0.77; 95%CI: 0.56–1.06; p=0.043)		
								CT-bevacizumab vs CT:		
								**mPFS** 6.8 m vs 5 m		
								(aHR: 0.70; 95%CI: 0.52–0.95; p=0.010)		
								**RR** 21% vs 17%		
								(p=0.573)		
	EAGLE UMIN000002557	R, OL, Phase III	mCRC, prior 1^st^-line bevacizumab + an oxaliplatin-based regimen	FOLFIRI-bevacizumab 5 mg/kg vs FOLFIRI- bevacizumab 10 mg/kg	1. ECOG PS (0 vs 1)	387	PFS	Bevacizumab 5 mg/kg vs 10 mg/kg:	Similar incidence of all-grade hematologic and non-hematologic AEs, including bevacizumab-related	(48)
					2. number of metastatic organs			**mPFS**		
					(<2 vs ≥2)			6.1 m (95%CI: 5.3–7.0) vs		
					3. reason for starting second-line treatment (PD vs toxicity)			6.4 m (95%CI: 5.6–7.4)		
					4. early recurrence within 6 m from adjuvant CT			(HR: 0.95; 95%CI: 0.75–1.21; p=0.676)		
								**median second PFS from the start of the 1^st^-line treatment to PD after receiving the study treatment:**		
								17.4 m (95%CI: 16.1–19.4) vs		
								17.6 m (95%CI: 16.0–18.9)		
					(yes vs no)			(HR: 1.00; 95%CI: 0.79–1.26; p= 0.976)		
					5. institution			**mTTF**		
								5.2 m (95%CI: 4.5– 5.8) vs		
								5.2 m (95%CI: 4.4–5.7)		
								(HR: 1.01; 95%CI: 0.81–1.25; p=0.967)		
								**PR** 11% vs 11%		
								**mOS**		
								16.3 m (95%CI: 14.1–21.2) vs		
								17 m (95%CI: 14.6–19.1)		
								(HR: 1.08; 95%CI: 0.75–1.57;		
								p=0.667)		
	ITACa	R, OL, Phase III	mCRC, pts previously untreated for advanced disease	Arm A: 1^st^-line CT-bevacizumab followed by 2nd-line CT alone	1. CT regimen	370	PFS	**Effect of bevacizumab across the two lines:** HR:0.80 (95%CI 0.68–0.95), p=0.008	1^st^-line G≥3 AEs	(49, 50)
				Arm B: 1^st^-line CT alone followed by 2nd-line CT-bevacizumab- or CT-bevacizumab-cetuximab	2. KRAS status (WT vs MT vs unknown)			**Addition of bevacizumab to 1^st^-line CT:** HR:0.90 (95%CI 0.72–1.12), p = 0.340 [i.e. 10% risk reduction]	Arm A vs B:	
								**Addition of bevacizumab to 2^nd^-line CT:** HR:0.64 (95%CI 0.49–0.84), p=0.0011 [i.e. 36% risk reduction]	Fatigue: 10.3% vs 3.15% (p=0.031)	
	PRODIGE18	R, OL, Phase II	mCRC, KRAS exon 2 WT; PD after bevacizumab + 5FU + irinotecan or oxaliplatin	Arm A: FOLFIRI or mFOLFOX6 + bevacizumab Arm B: FOLFIRI or mFOLFOX6 + cetuximab	1. 1^st^-line CT (fluoropyrimidine + oxaliplatin vs fluoro-pyrimidine + irinotecan)	132	4 m-PFS rate	Arm A (bevacizumab) vs Arm B (cetuximab):	G≥3 AEs	(51)
				(2^nd^-line CT regimen according to ding to 1^st^-line CT [i.e. crossover]).	2. first-line PFS (≤9 m vs >9 m)			**4-m PFS rate** 80.3% (95%CI, 68.0–88.3%) vs 66.7% (95%CI, 53.6–76.8%)	Arm A vs Arm B:	
					3. center			**mOS** 32.7 m (range 25.4–36.6) vs 25.5 m (range 21.8–34.8)	Diarrhea 7.7% vs 9%	
								(HR: 0.89; 95%CI: 0.58–1.35; p=0.58)	Fatigue 10.8% vs 10.4%	
								**mOS in KRAS WT, NRAS WT subgroup:** 36.3m (95%CI: 24.0–41.0) vs 24.8 m (95%CI: 21.0–36.0 m), p=0.56	Neutropenia 18.5% vs 14.9%	
								**mOS in KRAS WT, NRAS WT, BRAF WT subgroup:** 36.6** m** (95%CI: 20.1–45.6 m) vs 28.1 m (95%CI: 21.0–36 m), p=0.71	Arm B:	
									Stomatitis 7.5%	
									Anemia 13.4%	
									Skin disorders 19.4%	
	SPIRITT	R, OL, Phase II	mCRC (KRAS exon 2 WT); prior 1^st^-line oxaliplatin-based CT + bevacizumab (≥4 doses of the combination) and interrupted treatment due to PD or toxicity	FOLFIRI-panitumumab vs FOLFIRI-bevacizumab 5 or 10 mg/kg	1. 1st-line treatment failure (PD vs toxicity)	182	PFS	FOLFIRI-panitumumab vs FOLFIRI-bevacizumab:	G≥3 AE	(52)
					2. intended bevacizumab dose (5 vs 10 mg/kg)			**mPFS**	FOLFIRI-panitumumab vs FOLFIRI-bevacizumab:	
								7.7 m (95%CI 5.7–11.8) vs		
									Skin disorders 30% vs 2%	
								9.2 m (95%CI 7.8–10.6)	Neutropenia 23% vs 30%	
								(HR: 1.01; 95%CI: 0.68–1.50), p=0.97	Diarrhea 20% vs 9%	
								**mOS**	Hypomagnesemia 13%vs 0%	
								18 m (95%CI: 13.5–21.7) vs		
								21.4m (95%CI: 16.5–24.6)	Hypokalemia 14% vs 5%	
								(HR:1.06; 95% CI: 0.75–1.49), p=0.75	Dehydration 10% vs 4%	
								**ORR**	Hypotension 5% vs 0%	
								32% (95%CI: 23–43%)vs		
								19% (95%CI: 11–29%)		
Ramucirumab	RAISE	R, OL, Phase III	mCRC; with PD during or <6m of the last dose of 1^st^-line bevacizumab + oxaliplatin + pyrimidine	FOLFIRI-ramucirumab vs FOLFIRI-placebo	1. geographic region (North America vs Europe vs all other regions)	1072	OS	FOLFIRI-ramucirumab vs FOLFIRI-placebo:	G≥3 AEs FOLFIRI-ramucirumab vs FOLFIRI-placebo:	(44)
					2. KRAS exon 2 status (MT vs WT)			**mOS** 13.3 m vs 11.7 m	Neutropenia 38% vs 23%	
					3. TTP after start of 1st-line therapy (<6 m vs ≥6 m)			(HR: 0.84; p=0.0219)	Hypertension 11% vs 3%	
								**mPFS** 5.7 vs 4.5 m	Fatigue 12% vs 8%	
								(HR: 0.79; p=0.0005)		
Aflibercept	VELOUR	R, DB, Phase III	mCRC; PD during or after completion of a single prior oxaliplatin-containing regimen^3^	FOLFIRI-aflibercept vs FOLFIRI-placebo	1. prior treatment with bevacizumab (yes vs no)	1226	OS	FOLFIRI-aflibercept vs FOLFIRI-placebo:	FOLFIRI-aflibercept vs FOLFIRI-placebo:	(45)
					2. ECOG PS (0, 1, or 2)			**mOS** 13.5 m vs 12.1 m	AE any grade: Hypertension 41.4% vs 10.7%	
								(HR: 0.817; 95%CI: 0.713–0.937; p=0.0032)	Diarrhea: 69.2% vs 56.5% Nausea and abdominal pain 34% vs 29.1%	
								**mPFS** 6.9 m vs 4.7 m	Neutropenia 67.8% vs 56.3%	
								(HR: 0.758; 95%CI: 0.661–0.869; p<0.0001)	Asthenia 60.4% vs 50.2% Stomatitis: 54.8% vs 34.9%	
								**RR**	G≥3 AEs:	
								19.8% (95%CI: 16.4–23.2) vs	Neutropenia	
								11.1% (95%CI: 8.5–13.8)	G3: 23.1% vs 19.1%	
								p=0.0001	G4: 13.6% vs 10.4%	
									Hypertension	
									G3: 19.1% vs 1.5%	
									G4: 0.2% vs 0%	
									Diarrhea	
									G3: 19% vs 7.6%	
									G4: 0.3% vs 0.2%	
									Fatigue	
									G3: 16% vs 10.4%	
									G4: 0.8% vs 0.2%	
	NCT01661270	R, DB, Phase III	Asia –Pacific population with mCRC; PD during or after last administration of an oxaliplatin-containing regimen^4^	FOLFIRI-aflibercept vs FOLFIRI-placebo	1. ECOG PS	332 (ITT)^5^	PFS	FOLFIRI-aflibercept vs FOLFIRI-placebo:	FOLFIRI-aflibercept vs mixed administration^5^ vs FOLFIRI-placebo:	(53)
					(0 vs 1)			**mPFS**	G≥3 AEs	
					2. prior bevacizumab (yes vs no)			6.9 m (95%CI: 6.045–7.655) vs	Neutropenia	
								5.6 m (95%CI: 4.632–6.111)	29% vs 51% vs 27%	
								(stratified HR: 0.629; 95%CI: 0.488–0.812)	Diarrhea	
								**OS** aHR: 0.794; 95%CI: 0.606–1.042	17% vs 16% vs 9%	
								**ORR** 18% vs 4%		

^1^Single agent bevacizumab arm closed at an early interim analysis because of suggested inferior outcomes (41).

^2^Patient accrual to this study was stopped after TML results (43).

^3^Patients relapsed within 6 months of completion of oxaliplatin-based adjuvant therapy and bevacizumab pre-treated were also eligible (45).

^4^Patients who relapsed rapidly within 6 months of completion of oxaliplatin-based adjuvant chemotherapy were also eligible (53).

^5^Due to a clinical supply misallocation, 198 (60%) received at least one cycle of misallocated treatment (aflibercept or placebo; all still received FOLFIRI) (53).

1^st^-line, first-line; 2^nd^-line, second-line; 5-FU, fluouracil; AE, adverse event; aHR, adjusted HR; BRAF, v-raf murine sarcoma viral oncogene homolog B1; CI, confidence interval; CT, chemotherapy; DB, double-blind; EP, endpoint; FOLFOX, fluorouracil, levofolinic acid, oxaliplatin; FOLFIRI, fluorouracil, levofolinic acid, irinotecan; G, grade; HR, hazard ratio, ITT, intent to treat; KRAS, Kirsten rat sarcoma viral oncogene homolog; m, months; mCRC, metastatic colorectal cancer; mFOLFOX6, modified FOLFOX-6; mOS, median overall survival; mPFS, median progression free survival; MT, mutant; mTTF, median time to treatment failure; NRAS, neuroblastoma RAS viral oncogene homolog; OL, open-label; OS, overall survival; PD, disease progression; PFS, progression free survival; pts, patients; R, randomized; RR, response rate; vs, versus; WT, wild type. The underlined text and the bold values refer to some points that we would like to emphasize, e.g. endpoints, treatment arms.

Bevacizumab is a monoclonal antibody (mAb) targeting anti-VEGF-A; its role in the second-line setting evaluated both in the bevacizumab-naïve and bevacizumab-pretreated settings, and both in RAS wild type (WT) and mutant type (MT) patients. Treatment sequence strategies with bevacizumab as second-line treatment after first-line bevacizumab include maintenance therapy versus “stop and go” treatment, which were investigated in several studies (CAIRO3, AIO 0207, PRODIGE 9, OPTIMOX2 and SAKK 41/06 studies), a meta-analysis (including OPTIMOX2, CAIRO3 and AIO 0207 trials) (55), and a pooled analysis of randomized phase III trials (56). Maintenance therapy was associated with significantly improved time to failure (hazard ratio (HR) 0.79; 95% confidence interval (CI) 0.7–0.9, p=0.0005) (56) and progression-free survival (PFS) than “stop and go” [meta-analysis HR 0.53, 95%CI 0.40–0.69 (55); pooled analysis HR 0.56; 95%CI 0.44–0.71, p<0.00001 (56)]. Based on these data, the maintenance strategy appears appealing and aims to reduce the occurrence of oxaliplatin-induced neuropathy, although clinicians should expect to encounter other toxicities that are likely to eventually develop with such an approach, including hypertension and proteinuria.

Other treatment sequencing strategies investigated with bevacizumab include first-line chemotherapy plus bevacizumab followed by second-line chemotherapy alone (Arm A), which was compared with first-line chemotherapy alone followed by second-line chemotherapy plus bevacizumab (with or without cetuximab, according to KRAS status, Arm B) in the ITACa trial (49, 50) (**Table 2**). However, results from this study should be interpreted with caution because of significant study limitations, including slow and poor recruitment, a change in the primary objective (from OS to PFS), the high proportion of patient withdrawals and consequently a lack of patients entering into second-line treatment with or without bevacizumab, thus affecting the treatment duration. Indeed, patients in Arm A received a median of 12 chemotherapy cycles [range 1–43, interquartile range (IQR) 6–16] and among the 45 patients who received bevacizumab maintenance, treatment duration was restricted to a median of 6 cycles (range 1–30, IQR 3–13).

Ramucirumab is a fully human immunoglobulin (Ig) G-1 mAb which binds to the VEGFR-2 extracellular domain; it has been evaluated in combination with folinic acid, fluorouracil (5-FU), and irinotecan (FOLFIRI) as second-line treatment in patients pretreated with oxaliplatin, fluoropyrimidine, and bevacizumab (44) (**Table 2**).

Aflibercept, also known as VEGF-Trap, was evaluated in combination with FOLFIRI in the second-line setting in the prospective, international, randomized, double-blind, parallel-group phase III VELOUR study (45, 46) and in a phase III trial in an Asian patient population by Li and colleagues (53) (**Table 2**). Currently, aflibercept can be administered only in combination with a FOLFIRI backbone; mFOLFOX6 is an unsuitable backbone (no significant improvement in PFS, and very high toxicity, when administered in combination with mFOLFOX6 in the first-line setting in the AFFIRM trial) (57).

There is only evidence from phase I and II trials (58, 59) and no evidence from randomized studies for the use of aflibercept with capecitabine-based or 5-FU bolus-based treatment.

## Resistance Mechanisms

There are couple of known mechanisms whereby resistance to VEGF-targeted therapies can develop. Indeed, in most patients, despite prolonged anti-angiogenic treatment and VEGF-A blockade, angiogenesis is re-established. Anti-angiogenic agents also act by normalizing the blood vessel texture, which is convoluted and dysfunctional in cancer, thus increasing blood flow and suggesting that the administration of an anti-angiogenic drug plus chemotherapy increases the delivery of the chemotherapeutic agent to the cancer tissue (60). Preclinical studies showed the tumor capability to develop compensatory mechanisms leading to restoration of high vessel density and consequently cancer growth; this phenomenon appears related to hypoxia-triggered upregulation of alternative pro-angiogenic factors (19). The biomarker landscape in mCRC is evolving (61), but unfortunately to date, no biomarkers to define which patients will respond to anti-angiogenic treatment and who will develop resistance have been validated yet, although several have been investigated (tissue-based or circulating).

Various angiogenesis-related single nucleotide polymorphisms (SNPs) in the VEGF gene have been studied in mCRC (62–64). In 46 patients receiving first-line bevacizumab-based therapy, the CC genotype of rs3025039 polymorphism of VEGF-Ac.*237C>T was significantly associated with time to treatment failure; patients with at least one T allele had worse OS and PFS (64). Conversely, VEGF-A rs699947 A/A allele was associated with increased PFS and OS and the ICAM-1 rs1799969 G/A allele was correlated with longer OS (64). Worse OS with bevacizumab regimens was observed in patients with CD133 CC genotype in the rs3130 (65). Shorter OS and PFS with chemotherapy plus bevacizumab were reported for VEGFR1 rs9513070/rs9554320/rs9582036 GCA haplotype* (66) and BMAL1 SNPs (rs7396943, rs7938307, rs2279287) (67). An interleukin (IL) 8 polymorphism (c.-251TA+AA) (39) and the IL-6 rs2069837 G allele were associated with worse PFS with bevacizumab-based therapies (40). In the TME trial (ML18147), SNP analysis found a correlation between OS and VEGFA, VEGFR2 and EGLN1 SNPs; moreover, a correlation between PFS and VEGFA, VEGFR1, VEGFR2, EGLN3 SNPs was observed (68). Five HIF2α SNPs were associated with bevacizumab treatment effect.

Angiogenesis genotyping by Giampieri and colleagues in 138 mCRC patients treated with regorafenib, found that the VEGF-A rs2010963 SNP maintained an independent correlation with PFS and OS (69). Recent evidence suggests micro-RNA(miRNA) might modulate tumor angiogenesis by targeting anti- and pro-angiogenic factors, including hypoxia inducible factor (HIF), VEGF, and EGF. miR-23a-3p was reported as a key regulator of IL-17C-induced tumor angiogenesis in CRC (70), and high expression of miRNA-126 is related to increased VEGF-A signaling in endothelial cells and might be a promising biomarker for anti-angiogenic therapies (71). Nevertheless, data on the predictive role of miRNA remain preliminary.

Resistance to bevacizumab can result from the development of alternative angiogenesis pathways (72, 73). This induced pro-angiogenic factor substitution *via* activation and/or upregulation of members of the fibroblast growth factor (FGF) family, notch signaling, ephrin, or angiopoietin-1 re-establishes tumor neovascularization, resulting in tumor relapse (72, 73). In some patients, the angiogenic factor profile is different before the occurrence of progressive disease (PD) than that observed at the time of radiographic progression (74). Indeed, elevated levels of pro-angiogenic cytokines [e.g., basic FGF (bFGF)] placental growth factor (PlGF), hepatocyte growth factor (HGF)], have been demonstrated in subsets of patients prior to radiographic PD, with the suggestion that compensatory angiogenic factors may be stimulating new vessel growth in preparation for clinically evident progression (74). In such patients, second-line treatment with alternative anti-angiogenic therapies, rather than bevacizumab continuation, would be more beneficial (74).

Several circulating biomarker studies have been performed to evaluate the role of angiogenic factors other than VEGF-A in patients treated with anti-angiogenic agents (**Table 3**) **(** 43–45, 75–82). Recently, the role of PlGF emerged as another potential crucial factor involved in anti-angiogenic agent resistance. PlGF was initially considered only as an indirect actor of angiogenesis, more specifically as a competitor for VEGF-A to bind VEGFR-1 and soluble VEGFR-1 (sVEGFR-1) thus increasing the availability of VEGF-A to bind and activate VEGFR-2 (83). Conversely, PIGF can also directly induce angiogenesis through regulation of the crosstalk between VEGFR-1 and VEGFR-2, amplification of the VEGF-A signal through VEGFR-2 activation, enhancement of the angiogenic signal through the activation of the VEGFR-1/VEGFR-2 heterodimers *via* VEGF/PlGF heterodimers, impairment of dendritic cell maturation leading to immune suppression and promotion of the metastatic process by recruiting pro-angiogenic progenitor cells from the bone marrow to the tumor and the pre-metastatic niche, with consequent proliferation of metastatic cells (23–25). Various studies reported an increase of PlGF in the development of resistance, despite an initial decrease of VEGF-A levels (77, 79, 84), thus suggesting its potential role in tumor resistance. In a study by Lieu and colleagues, PlGF and VEGF-D were associated with resistance to bevacizumab-containing chemotherapy in mCRC (78). In mouse models targeting PIGF lead to a reduction of tumor growth (85, 86). Upregulation of PlGF appears as one of the main mechanism of resistance to angiogenesis-blockade and as a consequence, is a crucial potential therapeutic target for mCRC patients who have progressed on VEGF or VEGFR inhibitors (19). Bevacizumab targets only VEGF-A, preventing its interaction with VEGFR, so redundancy in angiogenesis pathways leads to treatment resistance *via* PlGF and VEGF-D. In contrast, aflibercept leads to trapping of VEGF-A, B, and PlGF-1 and PlGF-2, preventing VEGF and PlGF from binding to their native receptors, thus providing a more complete and efficient blockade of angiogenesis and its resistance strategies. Moreover, by inhibiting the upregulation of compensatory angiogenic factors, aflibercept may inhibit immune cell recruitment and further metastatic spread (28, 82). A *post hoc* biomarker analysis of the VELOUR trial assessed the impact of prior bevacizumab treatment and VEGF-A and PlGF levels on outcomes following second-line FOLFIRI-aflibercept treatment (**Table 3**) (82). In the FOLFIRI-aflibercept group, patients achieved prolonged OS and PFS irrespective of baseline VEGF-A and PlGF levels. So, aflibercept may provide benefit in patients with high VEGF-A or PlGF serum levels (82).

**Table 3:** Circulating pro-angiogenic biomarkers analysis.

Study/author	Treatment regimen	Setting	Findings	Reference
Giampieri et al. SENTRAL	FOLFIRI-bevacizumab	First-line	Changes in circulating FGF-2 levels among different blood samples seemed to correlate with clinical outcome	(75, 76)
			mPFS 12.8 m in pts whose FGF-2 levels increased at the second CT cycle compared with baseline vs 7.6 m in pts without FGF-2 increase (HR: 0.73; 95%CI: 0.43–1.27; p=0.23)	
			mPFS 12.9 m in pts whose FGF-2 levels increased between baseline and 8-week time point vs 8 m in pts without FGF-2 increase (HR: 0.78; 95%CI: 0.46–1.33; p=0.35)	
Kopetz et al.	FOLFIRI-bevacizumab	First-line	bFGF, PlGF, MMP-9, PDGF and HGF levels increase compared with baseline before radiographic PD (p < 0.001)	(74)
Loupakis et al.	FOLFOXIRI-bevacizumab	First-line	Biologically active VEGF concentration: prolonged and significant reduction during treatment, lower than baseline also at PD	(77)
			sVEGFR-2 and TSP-1 levels: increase at PD	
			PlGF levels: increase during treatment	
Lieu et al.	Discovery cohort: FOLFIRI-bevacizumab	discovery cohort: first-line; validation cohort: untreated or after PD on a first-line regimen ± bevacizumab	In the discovery cohort:	(78)
	Validation cohort: untreated pts or after PD on a regimen ± bevacizumab		VEGF-C levels increase prior to PD and at PD (+49% and +95%, respectively, p<0.01),	
			VEGF-D levels increase (+23%) at PD (p=0.05)	
			In the validation cohort:	
			Pts after PD on CT-bevacizumab: higher levels of PlGF (+43%, p=0.02) and VEGF-D (+6%, p=0.01) than untreated pts and significantly elevated levels of PlGF (+88%) than pts treated with CT alone	
			Transient elevations of PlGF and VEGF-D: back to baseline levels with a half-life of 6 weeks	
Horita et al. AVASIRI	FOLFIRI-bevacizumab	second-line	Decrease of VEGF-A serum concentration after the treatment start (p<0.0001)	(79)
			Increase of PlGF levels after the treatment start (p<0.0001)	
Cremolini et al. BEBYP	CT –bevacizumab vs CT	second-line	VEGFR-2 levels >6.3 ng/ml (n=30 pts): significant benefit in PFS when continuing bevacizumab:	(43, 80)
			**mPFS** 10.4 vs 3.4 m	
			(HR: 0.24; 95%CI: 0.10–0.58; p=0.002)	
			VEGFR-2 levels ≤6.3 ng/ml (n=29): no benefit from bevacizumab continuation	
			**mPFS** 5.4 m vs 5.0 m	
			(HR: 0.98; 95%CI: 0.45–2.11; p=0.956)	
			Significant interaction between VEGFR-2 levels and the effect of bevacizumab (p=0.036)	
Tabernero et al. RAISE Biomarker program	FOLFIRI-ramucirumab vs FOLFIRI-placebo	second-line	VEGF-D levels: statistical significance for OS and PFS confirmed by interaction analysis, 115 pg/ml cut-off (p=0.0005 and p<0.0001, respectively).	(44, 81)
			FOLFIRI-ramucirumab vs FOLFIRI-placebo:	
			High VEGF-D subgroup:	
			**mOS**	
			13.9 m (95% CI: 12.5–15.6) vs	
			11.5 m (95% CI: 10.1–12.4)	
			(stratified HR: 0.73; 95%CI: 0.60–0.89; p=0.0022)	
			**mPFS**	
			6 m (95% CI: 5.6–7.0) vs	
			4.2 m (95%CI: 4.1–4.5)	
			(stratified HR: 0.62, 95%CI: 0.52–0.74; p<0.0001)	
			Low VEGF-D subgroup:	
			**mOS**	
			12.6 m (95%CI: 10.7–14.0) vs	
			13.1 m (95%CI: 11.8–17.0)	
			(stratified HR: 1.32, 95%CI: 1.02–1.70; p=0.0344)	
			**mPFS**	
			5.4 m (95%CI 4.2–5.8) vs	
			5.6 m (95%CI 5.3–6.9)	
			(stratified HR: 1.16 (95%CI: 0.93–1.45); p=0.1930)	
Van Cutsem et al. VELOUR Biomarker post-hoc analysis	FOLFIRI-aflibercept vs FOLFIRI-placebo	second-line	VEGF-A, PlGF, endoglin, T-cad, VEGFR-3, SAP-component, VDBP, NRP1 and CRP implicated in angiogenesis or bevacizumab resistance correlated with prior bevacizumab therapy (p<0.01)	(45, 82)
			VEGF-A (p = 1×10^−58^) and PlGF (p = 2.8×10^−13^) levels were elevated at baseline in bevacizumab pre-treated patients	
			FOLFIRI-aflibercept arm:	
			Prolonged OS and PFS irrespective of baseline VEGF-A and PlGF levels	
			FOLFIRI-placebo arm:	
			High baseline VEGF-A levels (>144 pg/mL): worse OS and PFS vs lower levels (9.6 m vs 12.9 m and 4 m vs 5.5 m, respectively)	
			High baseline PlGF levels (>8 pg/mL): worse OS and PFS vs lower levels (9.7 m vs 11.7 m and 4 vs 5.3 m, respectively)	

CI, confidence interval; CRP, C-reactive protein; CT, chemotherapy; FGF-2, Fibroblast growth factor-2; FOLFOXIRI, fluorouracil, levofolinic acid, oxaliplatin, irinotecan; FOLFIRI, fluorouracil, levofolinic acid, irinotecan; HGF, hepatocyte growth factor; HR, hazard ratio; m, months; MMP-9, matrix metallopeptidase 9; mOS, median overall survival; mPFS, median progression free survival; n., number; NRP1, neuropilin-1; OS, overall survival; PD, disease progression; PDGF, platelet-derived growth factor; PlGF, placental growth factor; PFS, progression free survival; pts, patients; SAP, serum amyloid P; T-cad, T-cadherin; TSP-1, thrombospondin-1; VDBP, vitamin D–binding protein; VEGF, vascular endothelial growth factor; sVEGFR-2, soluble vascular endothelial growth factor receptor 2; vs, versus. The underlined text and the bold values refer to some points that we would like to emphasize, e.g. endpoints, treatment arms.

No definitive conclusions can be drawn on the plethora of data relating to putative biomarkers, and resistance mechanisms remain a multifactorial and challenging issue in mCRC.

## Individualized Treatment

While selected biomarker testing is now standard practice in CRC, the usefulness of other potential predictive and prognostic markers in clinical practice is unclear and still under evaluation, prompting the need for clear evidence-based recommendations. Currently, treatment algorithms, such as those developed by European Society for Medical Oncology and the Italian Medical Oncology Association (29, 31), provide guidance for the management of patients with mCRC according to RAS/BRAF status [wild type (WT) or mutant (MT)] and prior treatment.

### Second-Line Treatment for RAS MT mCRC Patients: Anti-Angiogenesis Beyond Progression

The continuation of anti-angiogenic blockade is now a standard option for mCRC patients who showed PD after first-line treatment with bevacizumab. Preclinical data suggested continuous expression of VEGF at PD occurrence and, as a consequence, a prolonged exposure to anti-angiogenic drugs might delay tumor growth (87). Some studies indicate that longer duration of anti-angiogenic treatment may lead to improved outcomes, whereas early discontinuation after first line chemotherapy could results in “tumor rebound” or the occurrence of more aggressive PD. Based on this rationale, anti-angiogenic blockade might continue to be effective even when tumor cells develop resistance to chemotherapy, while interruption of the anti-angiogenic inhibition could result in detrimental effects (39, 40). An exploratory analysis of the ML18147 trial assessed study outcomes according to Kirsten RAS oncogene (KRAS) status (47). Overall, 300/820 patients (49%) had KRAS MT tumors. In this group, mPFS was 5.5 months for patients receiving chemotherapy plus bevacizumab and 4.1 months for patients receiving chemotherapy only (HR: 0.70; 95% CI: 0.56–0.89; p = 0.0027), and median OS (mOS) was 10.4 versus 10.0 months, respectively (HR: 0.92; 95% CI: 0.71–1.18; p = 0.4969). In both analyses, no treatment interaction by KRAS status was observed (mPFS, p = 0.4436; mOS, p = 0.1266) suggesting that bevacizumab continuation might be an option beyond first progression, irrespective of KRAS status (47). Nevertheless, it is important to keep in mind that this trial excluded patients with aggressive disease (PD <3 months after the last bevacizumab administration, first-line PFS was <3 months, bevacizumab given for <3 months [consecutive] in the first-line setting) (42).

A *post hoc* analysis of the RAISE trial evaluated the association of RAS mutational status with outcomes. A favorable and comparable ramucirumab treatment effect was observed both for RAS MT (median OS 12.9 months with FOLFIRI-ramucirumab versus 11.5 months with FOLFIRI-placebo, HR: 0.86; 95% CI: 0.71–1.04, p = 0.1110; median PFS 5.7 months with FOLFIRI-ramucirumab versus 4.3 months with FOLFIRI-placebo, HR: 0.81; 95% CI: 0.68–0.97, p = 0.0209) and RAS/BRAF WT tumors (median OS 16.2 months with FOLFIRI-ramucirumab versus 15.5 months with FOLFIRI-placebo, HR: 0.86; 95% CI: 0.64–1.14, p = 0.2899; median PFS 5.7 months with FOLFIRI-ramucirumab and with FOLFIRI-placebo, HR: 0.78; 95% CI: 0.6–11.0, p = 0.0512). Treatment-by-mutation status interaction tests (OS, p = 0.523; PFS, p = 0.655) indicated that the ramucirumab benefit was not statistically different among the mutation sub-groups (88). As specified in the study design and the inclusion criteria, the RAISE trial enrolled patients progressing after first-line treatment with oxaliplatin and bevacizumab, so the efficacy and safety of the anti-angiogenic sequence bevacizumab-ramucirumab was established right in the phase III pivotal trial.

Wirapati and colleagues evaluated the impact of RAS, BRAF and sidedness on aflibercept activity in mCRC patients enrolled in the VELOUR study; next generation sequencing (NGS) data on molecular status were available for 482 of 1226 patients, and 264 patients had RAS MT disease (89). The treatment effects on OS for the 482 patients was confirmed significant (HR: 0.80; 95% CI: 0.65–0.99), and similar to the intention-to-treat (ITT) results (HR: 0.82; 95% CI: 0.71–0.93). RAS MT patients receiving FOLFIRI-aflibercept had an OS of 12.6 versus 11.2 months for those receiving FOLFIRI-placebo (HR: 0.93; 95% CI: 0.70–1.23 p = 0.13). None of the mutation subgroup results showed significant interaction, and sidedness didn’t influence efficacy (89).

Thus, aflibercept, bevacizumab and ramucirumab show potential benefit in the treatment of RAS MT mCRC patients. Bevacizumab and aflibercept have been compared in this setting in an Italian, real-world, retrospective, single-center, non-randomized study (90). Seventy-four RAS MT mCRC patients whose disease had progressed after first-line treatment with FOLFOX-bevacizumab received second-line FOLFIRI-bevacizumab (arm A) or FOLFIRI-aflibercept (arm B). The two regimens appeared equally effective; despite a longer mOS observed for the combination of FOLFIRI-aflibercept, statistical significance was not reached (12.1 vs 8.9 months; HR: 1.02; 95% CI: 0.57–1.84). The study presented several biases which might have influenced results: in the FOLFIRI-aflibercept arm patients had a more extensive disease (>2 metastatic sites), a significant shorter duration of first-line treatment, and no maintenance treatment was allowed (90). Also, given the retrospective nature of the research, these data should be interpreted with extreme caution.

### Second-Line Treatment for RAS WT mCRC Patients – Focus on Sequence Anti-EGFR – Anti-Angiogenic Agents

All anti-angiogenic agents approved in second-line setting demonstrated their efficacy in RAS WT patients (47, 88, 89, 91), regardless of prior treatment with anti-angiogenic drugs. The administration of two consecutive lines including an anti-angiogenic agent has already been discussed in section 4.1 (both in RAS MT and WT patients); for these patients, the subsequent use of an anti-EGFR mAb in further lines remains an option.

Currently, second-line options for RAS WT mCRC patients progressing after first-line chemotherapy and an anti-EGFR mAb include bevacizumab and aflibercept (29–31, 92). To date, limited data are available on mCRC patients receiving aflibercept after an anti-EGFR based-treatment and no head-to-head comparative trials have been conducted to assess which is the best anti-angiogenic agent in this specific treatment setting. As a consequence, and clinical practice is essentially based on speculations deriving from first-line studies (93, 94). The retrospective SLAVE study evaluated the effectiveness of second-line bevacizumab-based or aflibercept-based regimens in 277 RAS WT mCRC patients progressing after a first-line anti-EGFR based treatment in a multicenter real-world cohort (95). No statistically significant difference between patients receiving bevacizumab-based and those receiving aflibercept-based regimens was observed in univariate analyses of objective response rate (ORR), PFS (HR: 1.34; 95% CI: 0.95–1.89; p = 0.0932) and OS (HR: 1.31; 95% CI: 0.89–1.93; p = 0.1600). As for multivariate analysis, after adjusting for the key covariates (age, gender, performance status, number of metastatic sites, and primary tumor side) bevacizumab-based regimens had slightly longer PFS than aflibercept-based regimens (HR: 1.44; 95% CI: 1.02–2.03; p = 0.0399), whereas no significant difference in OS was observed (HR: 1.47; 95% CI: 0.99–2.17; p = 0.0503). These data must be considered with caution due to the retrospective nature of the study, therefore no definitive conclusions can be drawn (95). In another retrospective study, Vera and colleagues analyzed the efficacy and safety of second-line FOLFIRI-aflibercept in RAS WT mCRC patients resistant to, or who had progressed after, an oxaliplatin plus anti-EGFR regimen (96). PFS was 6.9 months (95% CI: 6.1–7.8), the ORR was 33% and mOS was 14.5 months (95% CI: 9.7–19.3). As for safety, 37.5% of the patients reported grade 3–4 toxicities (hematologic 16.6%, hypertension 7.5%, asthenia 5.9%, and perforation 2.5%). Though retrospective, these results were consistent with the VELOUR trial, showing FOLFIRI-aflibercept efficacy was maintained irrespective of prior anti-EGFR treatment, thus suggesting a role for this regimen also in this population (96). The ongoing, prospectively stratified, biologically enriched, multicenter, phase II DISTINCTIVE study is assessing the efficacy of aflibercept in combination with FOLFIRI in the second-line treatment of RAS WT mCRC patients who received first-line oxaliplatin in combination with an anti-EGFR mAb (either panitumumab or cetuximab); one of the study aims is to prospectively validate VEGFR2 plasma levels as a predictive factor for efficacy of aflibercept in combination with FOLFIRI (97).

In the FIRE-3/AIO KRK0306 trial, evidence seemed to suggest that in WT patients the sequence anti-EGFR–anti-angiogenic might lead to more favorable results than the reverse sequence. Indeed, both PFS (6.5 vs 4.7 months; HR: 0.68; 95% CI: 0.54–0.85; p < 0.001) and OS (16.3 vs 13.2 months; HR: 0.70; 95% CI: 0.55–0.88; p = 0.0021) from start of second-line treatment were longer in patients treated with oxaliplatin-based chemotherapy plus bevacizumab after FOLFIRI plus cetuximab versus oxaliplatin-based chemotherapy plus cetuximab or panitumumab after FOLFIRI plus bevacizumab (HR: 0.95; 95% CI: 0.55–1.63; p = 0.841). In the CALGB/SWOG 80405 trial comparing first-line therapy with cetuximab vs bevacizumab plus mFOLFOX6 or FOLFIRI, 88% of patients received subsequent therapy, but no detailed or specific information on second-line treatment is available yet to determine the impact of treatment sequence on survival parameters. Further data from larger prospective trials in RAS WT mCRC patients focused on second-line treatment are needed to assess the best treatment option in this setting. Indeed, to date, the major of data are derived from patients enrolled in the CALGB/SWOG 80405 and FIRE-3 trials and who had received second-line treatment; no prospective specific second-line studies have been performed in this patient setting.

### Second-Line Treatment for BRAF MT mCRC Patients

BRAF mutation is present in 5–10% of mCRC patients and its correlation with poor prognosis widely known (98, 99). Unfortunately, survival after disease progression on first-line chemotherapy is markedly shorter in BRAF MT than WT patients (4.2 vs 9.2 months, adjusted HR: 1.69; p < 0.001), and fewer MT patients go on to receive second-line therapy (33% vs 51%), either because they are ineligible (unfit) or because their disease progresses so rapidly (100). In the exploratory analysis of the ML18147 trial, only 14 patients had a BRAF mutation (7%, 6 patients in the bevacizumab plus chemotherapy group and 8 in the control group) and due to the very small number of BRAF MT subjects no correlative analysis could be carried out (47). In the *post hoc* analysis of the RAISE trial, 41 patients (4.5%) were BRAF MT. Ramucirumab-treated BRAF MT patients showed a promising trend in OS and PFS benefit with ramucirumab over placebo, with mOS and mPFS more than double that of placebo (mOS, 9.0 vs 4.2 months; HR: 0.54; 95% CI: 0.25–1.13; mPFS, 5.7 vs 2.7 months; HR: 0.55; 95% CI: 0.28–1.08). BRAF MT mCRC patients had worse survival than RAS/BRAF WT regardless of treatment, confirming BRAF as a negative prognostic factor also in the second-line setting. The sample size was too small to draw definitive conclusions on real difference of ramucirumab effect in the BRAF MT patients versus the RAS/BRAF WT or RAS MT, and requires further validation (88). Even if none of the mutation subgroups demonstrated significant interaction and though limited by the small sample size, the VELOUR analysis showed also a promising trend for better outcome with aflibercept and FOLFIRI for BRAF MT mCRC patients in OS, PFS and RR. Globally, 36 patients harboring BRAF MT were evaluated; mOS was 10.3 months in the FOLFIRI-aflibercept group versus 5.5 months in the control group (HR: 0.42; 95% CI: 0.16–1.09; p = 0.08) and mPFS was 5.5 versus 2.2 months (HR: 0.59; 95% CI: 0.22–1.58) (89, 96). Gelsomino and colleagues conducted a pooled analysis with the aim to assess the impact of anti-angiogenic drugs in patients with pre-treated BRAF MT mCRC. The analysis included patients enrolled in randomized, controlled trials who received second-line chemotherapy plus either antiangiogenic agents, namely ramucirumab or aflibercept, or placebo. The results were then pooled with the data and outcomes of BRAF MT patients enrolled in TRIBE and TRIBE-2 study who had received either second-line chemotherapy plus bevacizumab or chemotherapy alone. This analysis included 129 patients and confirmed a significant advantage of anti-angiogenic drugs compared to placebo in terms of OS (HR: 0.50; 95% CI: 0.29–0.85; p = 0.01) in pre-treated BRAF MT mCRC patients.

Recently, the randomized, phase III, open-label BEACON CRC trial assessed the superiority in OS, ORR and patient-reported QoL of encorafenib plus cetuximab with binimetinib (triplet arm) or without binimetinib (doublet arm) versus either cetuximab-irinotecan or cetuximab-FOLFIRI (control arm) in BRAF V600E MT mCRC patients with PD after one (65%) or two previous regimens. Respective ORRs were 26.8% (95% CI: 21.1–33.1), 19.5% (95% CI: 14.5–25.4), and 1.8% (95% CI: 0.5–4.6). mOS was 9.3 months (95% CI: 8.2–10.8) in the triplet arm and 5.9 months (95% CI: 5.1–7.1) in the control arm (HR: 0.60; 95% CI: 0.47–0.75). mOS in the doublet arm was 9.3 months (95% CI: 8.0–11.3; HR vs control: 0.61; 95% CI: 0.48–0.77) (101–103).

Targeted therapy with encorafenib plus cetuximab is now an established second-line strategy for this subgroup of patients. The combination of chemotherapy plus bevacizumab, ramucirumab or aflibercept represents an alternative option, even if none of the chemotherapy-based combinations have been formally compared with encorafenib plus cetuximab (104, 105).

In the era of precision medicine and target-tailored treatment, on the basis of these recent findings in BRAF MT mCRC patients, the anti-angiogenic second-line approach and its direct comparison with second-line target doublet or triplet in this specific population surely requires further research.

## Focus on Aflibercept

Aflibercept, an innovative anti-angiogenic agent, is a recombinant fusion protein containing VEGF-binding portions from the extracellular domains of human VEGFR 1 and 2, fused to the Fc portion of human IgG1. Targeting VEGF-A, VEGF-B, and PlGF with high-affinity, aflibercept prevents these ligands from binding to their endogenous receptors and thus providing a wider pharmacologic blockade of the VEGF pathway (28, 45, 46). In this way, aflibercept is able to overcome biologic mechanisms of resistance occurring during previous angiogenesis blockade (28). In this section, we focus specifically on this innovative agent.

### VELOUR Trial – Exploratory and *Post Hoc* Analysis

Further analyses of the VELOUR study have been conducted to assess the efficacy and safety of aflibercept in specified populations and according to the molecular profile of mCRC.

#### Survival

An integrated analysis of the time courses of both the efficacy and safety of aflibercept plus FOLFIRI confirmed a continued and persistent OS increase over time. Indeed, mOS improved progressively to 2.6 months at 18 months and 4.4 months at 24 months. The estimated probabilities of survival were 38.5% versus 30.9% at 18 months, 28.0% versus 18.7% at 24 months and 22.3% versus 12.0% at 30 months for patients receiving FOLFIRI-aflibercept versus those receiving FOLFIRI-placebo, respectively, with a consistent proportional improvement in the HR over time; survival at 24 months was improved by 50% and almost doubled at 30 months. Notably, survival results were not influenced by post-VELOUR anti-cancer treatments. As for safety, even if most chemotherapy- and anti-VEGF-related grade 3–4 adverse events (AEs) were more common in patients receiving FOLFIRI-aflibercept versus FOLFIRI-placebo, they were reported within the first four treatment cycles, were mostly reversible and of single occurrence and decreased over further cycles. This information provides useful data to anticipate and treat drug-related toxicities (106).

Van Cutsem and colleagues conducted a *post hoc* survival analysis after the exclusion of patients who had disease recurrence during or within 6 months of completing adjuvant oxaliplatin-based therapy, namely the adjuvant rapid relapsers (ARR) (10% of patients; n = 124, including 17 patients who also received bevacizumab in the adjuvant setting) (46). Results showed that OS in the ITT minus ARR (ITT-ARR) population (n = 1102) was longer in the experimental arm than in the control arm (HR: 0.78; 95% CI: 0.68–0.90; median survival difference: 1.87 months). Moreover, in the subgroup of patients assigned to the prior bevacizumab stratum at randomization, OS was numerically longer if they received aflibercept plus FOLFIRI than placebo plus FOLFIRI (HR: 0.81; 95% CI: 0.63–1.04; median survival difference: 2.14 months). The benefit observed from aflibercept plus second-line FOLFIRI was irrespective of the timing of first-line PD (<3 months, ≥3 to <6 months, ≥6 to<9 months and ≥9 months), suggesting efficacy also in patients who rapidly progressed on first-line treatment. No unexpected toxicity occurred. Even if no definitive conclusion for the “pure second line setting” can be established, the comparison of this *post hoc* analysis with the VELOUR primary analysis suggests that the inclusion of the ARR may have underestimated the aflibercept benefit both in bevacizumab-pretreated and bevacizumab-naïve patients, and that subjects developing rapid progression after first-line treatment are good candidates for FOLFIRI-aflibercept (46).

#### Subgroups and Specific Populations Analysis

Pre-specified subgroup analyses of the VELOUR trial are shown in **Table 4**. In the analysis by Tabernero and colleagues, the efficacy of FOLFIRI-aflibercept over FOLFIRI-placebo was confirmed irrespective of demographic and baseline characteristics and stratification factors (107). Notably, a significantly greater benefit was observed with aflibercept for patients with liver-only metastases than those with either no liver metastases or ‘liver and other sites’ metastases (OS, p = 0.090; PFS, p = 0.008), thus suggesting aflibercept plus FOLFIRI as an optimal treatment choice in patients with liver-limited disease. Moreover, aflibercept efficacy was not decreased, and toxicity was not worsened, by previous exposure to an anti-angiogenic drug, showing aflibercept as an optimal candidate in this setting of patients (107).

**Table 4:** Pre-specified analysis of the VELOUR trial.

Analysis	Objectives	Variables	Results	Reference
Pre-specified subgroup analysis	Assessment of treatment effect in specific subgroups	Demographic characteristics:	No significant treatment interaction between FOLFIRI-aflibercept vs FOLFIRI-placebo and factors for both OS and PFS	(107)
		Age <65/≥65 years	Treatment effect favored aflibercept over control (HR: <1.0) for OS and PFS in all subgroups	
		Male/female		
		Caucasian-white/other		
		Western Europe/Eastern Europe/North America/South America/Other countries		
		Baseline characteristics:	Consistent treatment effect in favor of the aflibercept for OS and PFS for all subgroups	
		Prior/no prior hypertension	Significantly greater aflibercept benefit in case of liver-only metastases vs no liver metastases or liver and other sites metastases: p=0.090 for OS; p=0.008 for PFS	
		Number of organs with metastasis ≤1/>1		
		No liver metastasis or liver and other		
		metastasis/liver metastasis only		
		Colon-rectosigmoid-other/rectum		
		Stratification factors:	A difference in favor of aflibercept over placebo in OS and PFS in each stratification subgroup; no significant interaction at the two-sided 10% level between treatment and stratification levels → no evidence of heterogeneity in treatment effect	
		ECOG PS	ECOG PS 0 vs 1 vs 2: p=0.7231 for OS; p=0.6954 for PFS	
		Prior bevacizumab	Prior bevacizumab (30.4% of ITT) vs no prior bevacizumab: p=0.5668 for OS; p=0.1958 for PFS	
			FOLFIRI-aflibercept vs control:	
			**mOS**	
			Prior bevacizumab: 12.5 m vs 11.7 m	
			No prior bevacizumab: 13.9 m vs 12.4 m	
			**mPFS**	
			Prior bevacizumab: 6.7 m vs 3.9 m	
			No prior bevacizumab: 6.9 m vs 5.4 m	
			No evidence of greater toxicity in pts previously treated with bevacizumab	
Pre-specified *post hoc* multivariate analysis of the ITT population	- Identification of prognostic factors associated with improved OS with FOLFIRI-aflibercept	Better efficacy subgroup:	Better efficacy subgroup FOLFIRI-aflibercept vs FOLFIRI-placebo:	(108)
	-Primary endpoint: OS in the better and poorer efficacy patient subgroup identified in the multivariate analysis	ECOG PS 0 with any number of metastatic site or ECOG PS 1 with <2 metastatic sites	Interaction with treatment: p = 0.0147 → differential OS effect of aflibercept compared with placebo	
		Poorer efficacy subgroup:	**mOS** 16.2 m (95% CI: 14.5–18.1) vs 13.1 m (95% CI: 11.7–14.2) (absolute difference in mOS: 3.1 m; aHR: 0.73; 95%CI: 0.61–0.86)	
		ECOG PS 1 with ≥2 metastatic sites or ECOG PS 2 with any number of metastatic sites or ARR (excluded from the analysis)	Continuous increase over time of the entity of survival differences between the two treatment arms: absolute OS rate difference 5% at 6 m →15% at 30 m	
			**mPFS** 7.2 m (95%CI: 6.8–8.2) vs 4.8 m (95%CI: 4.2–5.4)(absolute difference: 2.4 m)	
			Absolute difference in 6-month PFS rates: 25%	
			**ORR** 23.7% (95% CI: 19.3–28.2) vs 11% (95% CI: 7.8–14.3)	
			Poorer efficacy subgroup FOLFIRI-aflibercept vs FOLFIRI-placebo:	
			No OS, PFS and ORR improvement with FOLFIRI-aflibercept vs FOLFIRI-placebo	
			**mOS**	
			9.6 m (95% CI: 8.6–11.5) vs	
			10.4 m (95% CI: 9.5–12.1)	
			(aHR: 0.97; 95% CI: 0.78–1.21)	
Aged-based analysis	Assessment of benefit and safety of aflibercept in association with FOLFIRI according to age	Age ≥65 y	No treatment interaction between treatment group and age was reported for OS (p=0.683) or PFS (p=0.930)	(109)
		(36%; 84% aged 65–74 y, 97% ECOG PS 0–1)	≥65 y FOLFIRI-aflibercept vs FOLFIRI-placebo:	
		Age <65 y (64%)	**mOS** 12.6 m vs 11.3 m	
			(HR: 0.85; 95.34%CI: 0.68–1.07), absolute difference: 1.3 m	
			**mPFS** 6.6 m vs 4.4 m	
			(HR: 0.75; 99.99% CI: 0.48–1.17), absolute difference: 2.2 m	
			<65 y FOLFIRI-aflibercept vs FOLFIRI-placebo:	
			**mOS** 14.5 m vs 12.5 m	
			(HR: 0.80; 95.34%CI: 0.67–0.95)	
			**mPFS** 6.9 m vs 4.9 m	
			(HR: 0.77; 99.99%CI: 0.55–1.08), absolute difference 2 m	

AEs, adverse events; aHR, adjusted HR; CI, confidence interval; ECOG PS, Eastern Cooperative Oncology Group Performance Status; FOLFIRI, fluorouracil, levofolinic acid, irinotecan; G, grade; HR, hazard ratio, ITT, intent to treat; m, months; mOS, median overall survival; mPFS, median progression free survival; ORR, objective response rate; OS, overall survival; PFS, progression free survival; y, years. The underlined text and the bold values refer to some points that we would like to emphasize, e.g. endpoints, treatment arms.

The pre-specified *post hoc* multivariate analysis of the VELOUR ITT population, by Chau and colleagues, suggests that patients with an Eastern Cooperative Oncology Group Performance Status (ECOG PS) score of 0 and any number of metastatic sites and patients with ECOG PS of 1 and <2 metastatic sites might obtain a greater benefit from treatment with FOLFIRI-aflibercept, and this may help to improve the selection of patients (108).

An age-based analysis confirmed the efficacy of FOLFIRI-aflibercept in patients aged ≥65 and <65 years, despite an increase of AEs in older patients. However, through careful follow-up for toxicity and prompt management of AEs, mCRC patients with good PS may gain PFS and OS benefit from aflibercept in combination with FOLFIRI, irrespective of age, with previous evaluation and accurate selection of elderly patients (109).

### Aflibercept in Real-Life Setting

Aflibercept has been studied in real-life and clinical practice settings.

The prospective, observational, non-comparative, post-authorization safety study (PASS) OZONE trial conducted in 12 European and North American countries evaluated the safety and effectiveness of aflibercept plus FOLFIRI in 766 mCRC patients treated in daily practice after PD on an oxaliplatin-based regimen. 58.6% had received bevacizumab. Grade ≥3 treatment emergent AEs (TEAEs) were reported in 68.3% of patients, with neutropenia (15.1%), hypertension (10.2%), diarrhea (9.5%), and asthenia (9.1%) the most frequent, whereas AEs typically related to anti-angiogenic treatment were uncommon. No difference in the safety profile was observed in subgroup analyses except a more frequent incidence of grade ≥3 hypertension in bevacizumab-naïve patients. mOS was 12.5 months, mPFS was 6.1 months and ORR was 16.3%. Multivariate analysis found no statistical and clinically meaningful differences between groups defined by age, ethnicity, baseline renal function, or number of prior lines, even if the subgroups of patients without hepatic impairment and no prior use of bevacizumab seemed to be favored (110).

Several retrospective and real-world studies in mCRC patients receiving FOLFIRI-aflibercept have been performed in Spain (111–117), France (118–120), the USA (121) and Asia (122–126). Its safety profile described in these registry and real-world studies was consistent with those reported in clinical trials; even grade ≥3 AE rates were lower in the real-life population (neutropenia: 7.7–16.2%, fatigue 6–18%, hypertension 5.6–8%), although this finding might be related to underreporting or to an improved management of patients and AEs (127). The Aflibercept Safety and health-related Quality-of-life Program (ASQoP) (NCT01571284) was a global, multicenter, single-arm, open-label study evaluating the safety and health-related QoL (HRQoL) of FOLFIRI-aflibercept in mCRC patients previously treated with an oxaliplatin-containing regimen. The primary objective was to evaluate the safety of this therapeutic association in pre-treated mCRC patients in a setting more similar to real-life and the secondary objective was to assess the impact of this combination on patient-reported HRQoL. Moreover, this trial provided access to aflibercept to mCRC patients before marketing authorization and commercial availability. ASQoP enrolled 779 patients; FOLFIRI-aflibercept was well tolerated and the most common TEAEs of any grade were diarrhea (61.6%), hypertension (48.4%), and nausea (43.3%), whereas the most common grade 3–4 TEAEs were hypertension (24.1%), neutropenia (23.1%), and diarrhea (15.3%). The incidence of TEAEs was similar in bevacizumab pre-treated and bevacizumab-naïve patients (except grade 3–4 hypertension and any-grade proteinuria, with a slightly lower incidence in bevacizumab pre-treated patients), and in patients aged <65 and ≥65 years (aside from dehydration, which was more common in elderly than younger patients). No new safety signals emerged (128, 129). Clinically meaningful improvements and/or maintenance of HRQoL was reported in most patients (129). Also, in a cohort of 200 Italian patients from the study, no negative effects on HRQoL were observed and rates of TEAEs were similar to those reported in the VELOUR trial (128). These results were confirmed by an interim analysis of the larger (n = 1500 patients) ongoing QoLiTrap (AIO-LQ-0113) study, in which no clinically relevant deterioration in global health status evaluated through the EORTC-QLQ C30 questionnaire was observed during study treatment (130).

### Exploring Further Prognostic and Predictive Clinical and Translational Biomarkers

Montes and colleagues conducted an exploratory analysis of an observational, retrospective study in a real‐world population of 78 mCRC patients treated with FOLFIRI‐aflibercept as second‐line treatment or after rapid progression during adjuvant oxaliplatin with the aim to identify prognostic and predictive factors for survival outcomes. Regarding prognostic factors, metachronous versus synchronous metastasis and left versus right tumors were significantly related to survival. Patients who developed metachronous metastasis had significantly longer PFS (11.0 months; 95% CI: 4.1–17.9) compared with patients with synchronous metastasis (5.0 months; 95% CI: 3.0–7.0; p = 0.028); the same was observed for OS, which reached 17 months (95% CI: 7.8–26.2) in metachronous versus 10 months (95% CI: 8.2–11.8) in synchronous patients (p = 0.039). Moreover, mPFS was significantly longer in patients with left‐sided tumors (7 months; 95% CI: 5.2–8.8) versus 3 months (95% CI: 0.1–5.9) in patients with right‐sided tumors (p = 0.044); mOS was 12.0 months (95% CI: 9.9–14.9) in the left-sided group versus 8.0 months (CI 95%: 5.70–10.3) for the right-sided (p = 0.041). With regard to predictive factors, the occurrence of hypertension during treatment was related to significantly longer mPFS (10.6 months; 95% CI: 6.3–13.7 vs 4.0 months; 95% CI: 2.7–5.3) compared with patients who did not develop hypertension (p = 0.009) and OS (17.0 months; 95% CI: 0–35.5 vs 10.0 months; 95% CI: 7.2–12.8; p < 0.001). Furthermore, the study confirmed the efficacy and safety of FOLFIRI-aflibercept in a real-world population. This analysis is limited by the small number of patients enrolled and, therefore, the findings have to be interpreted with caution, particularly those of the timing of metastatic disease occurrence (113). The same authors developed and internally validated a prognostic nomogram in a multicenter sample of 250 patients from nine Spanish hospitals in order to stratify patients eligible for second-line FOLFIRI-aflibercept based on their probability of survival and to optimize treatment results. The prognostic nomogram for OS included six variables: ECOG PS, tumor location, number of metastatic sites, mutational status, better response to previous treatment, and carcinoembryonic antigen. The model was well calibrated and had acceptable discriminatory capacity (optimism-corrected c-index: 0.723; 95% CI: 0.666–0.778). mOS was 6.1 months (95% CI: 5.1–8.8), 12.4 months (95% CI: 9.36–14.8), and 22.9 months (95% CI: 16.6–not reached) for high-, intermediate-, and low-risk groups, respectively. Prognosis was not influenced by age, comorbidity, or use of modified FOLFIRI regimens (117).

Hamaguchi and colleagues conducted an ancillary exploratory analysis of the relationship between 78 potential prognostic biomarkers and efficacy endpoints following aflibercept plus FOLFIRI in 62 Japanese patients enrolled in a single arm, phase II study (125, 131). Baseline levels of extracellular newly identified receptor for advanced glycation end‐products binding protein (EN-RAGE), insulin‐like growth factor‐binding protein 1, IL-8, kallikrein 5, pulmonary surfactant‐associated protein D, tissue inhibitor of metalloproteinases 1 (TIMP-1), tenascin‐C, and tumor necrosis factor receptor 2 were correlated with OS in a univariate Cox regression analysis. The most significant OS differences were observed for TIMP-1, IL-8, and EN-RAGE (all p < 0.001); lower baseline concentrations of each of these were related to longer OS. Conversely, no correlation was found for PFS and maximum tumor shrinkage. Among the biomarkers having a ±30% change in plasma concentration from baseline to pre‐dose 3, P1GF was reported to have the most significant change (4716% change). In patients stratified by prior bevacizumab, baseline levels of log‐transformed VEGF, PlGF, and decorin were significantly higher in bevacizumab-pretreated patients; on the contrary, baseline levels of ANG‐2 were significantly lower in this population (131).

## Conclusions

Targeting angiogenesis is an effective strategy in the management of mCRC. Development of resistance and the discovery of various prognostic and predictive biomarkers require further considerations for the choice of second-line therapy.

In bevacizumab-naïve patients both bevacizumab or aflibercept (for patients progressing after an oxaliplatin-based regimen) represent second-line treatment options (29, 31), whereas bevacizumab, aflibercept or ramucirumab (in combination with FOLFIRI in patients who also received first-line oxaliplatin), might be an option in bevacizumab-pretreated patients (29, 31). Either aflibercept or ramucirumab, in combination with FOLFIRI, is specifically recommended in patients who progress quickly after first-line bevacizumab (29, 31). Ramucirumab is an option only after both oxaliplatin and bevacizumab. Aflibercept is effective and well tolerated, both in clinical trials and in real-life populations and represents a useful second-line strategy in combination with FOLFIRI in patients progressing after oxaliplatin-based chemotherapy, as well as in those with resistance after bevacizumab.

Each anti-angiogenic drug has its peculiar mechanism of action and demonstrated efficacy and safety in pivotal and real-world studies. Despite all the studies conducted so far, we do not yet have any validated biomarker which might guide our choice of second-line anti-angiogenic drug. The identification of pro-angiogenic plasma biomarkers would allow for selection of patients who would derive more benefit from second-line angiogenesis inhibition. Nonetheless, even if promising, preliminary findings cannot currently be applied to clinical practice. The lack of identification of a reliable biomarker might have various explanations. Firstly, angiogenesis is not a static but a dynamic process, with continuous changes and interactions among the different circulating angiogenic factors and the pathways involved, making identification of a single biomarker difficult. Moreover, defining and validating a quantitative biomarker threshold that is clearly associated with a benefit/resistance from available anti-angiogenic agents represents another challenge. Secondly, several factors contribute to the efficacy of a drug, so only angiogenesis by itself might not explain the global therapeutic results of an anti-angiogenic agent, thus making it difficult to identify a single robust factor as a biomarker.

While we await further data coming from clinical and translational studies that might guide biomarker-driven anti-angiogenic treatment, choice of second-line antiangiogenic drug currently has to be individualized for each patient according to their clinical features, outcomes and tolerability of prior treatments, and the tumor molecular profile.

## Author Contributions

EL, SC, and MS have contributed to the manuscript preparation, and have read and approved all drafts. All authors contributed to the article and approved the submitted version.

## Funding

Springer editorial assistance and publication cost were supported by an unrestricted grant from Sanofi Genzyme Italy. This medical writing and editorial assistance was funded by Sanofi Genzyme Italy. The funder was not involved in the study design, collection, analysis, interpretation of data, the writing of this article or the decision to submit it for publication.

## Conflict of Interest

SC has received speaker fees and grants from Amgen, Sanofi, Servier outside this study. MS has received consultant, advisory board and speakers’ bureau fees from Amgen, Sanofi, MSD, Eisai, Merck, Bayer outside this study.

The remaining author declares that the research was conducted in the absence of any commercial or financial relationships that could be construed as a potential conflict of interest.
